# Nonoperative management of a sagittal coracoid fracture with a concomitant acromioclavicular joint separation

**DOI:** 10.4103/0973-6042.70823

**Published:** 2010

**Authors:** Kristen Thomas, Vincent Y Ng, Julie Bishop

**Affiliations:** Sports Medicine Center, The Ohio State University, Columbus, OH 43221, USA

**Keywords:** Acromioclavicular joint, coracoid fracture, nonoperative treatment

## Abstract

Separation of the acromioclavicular joint in conjunction with a coracoid fracture is a rare injury. Treatment decisions are traditionally based on the level of the fracture, the status of the coracoclavicular ligament and the activity level of the patient. We present a novel coracoid fracture pattern treated nonoperatively in a young, active patient and a thorough review of the literature regarding this topic.

## INTRODUCTION

A fracture of the coracoid process associated with a complete acromioclavicular dislocation is rare, with only about 20 reported cases in adults in the English literature. Selecting the appropriate treatment for this injury may be difficult due to the varying treatment options reported in the literature. Although most authors recommend surgical treatment for these injuries and reserve nonsurgical management for older, sedentary patients or patients with medical comorbidities, there are several reports of young, active patients treated nonoperatively.[1] Choosing the proper treatment for a fracture of the coracoid process with a concomitant AC separation relies on successfully determining the stability pattern of the injury, which is largely dependent on the location of the coracoid fracture.

We present an athletic 22-year-old female with a type III AC separation and a unique coracoid fracture. Although her coracoid fracture pattern does not fit into the current classification system, it is believed to be a stable fracture pattern and was successfully treated nonoperatively. This report adds another case to the literature and reemphasizes the importance of recognizing this unusual lesion.

## CASE REPORT

**Figure 1:**
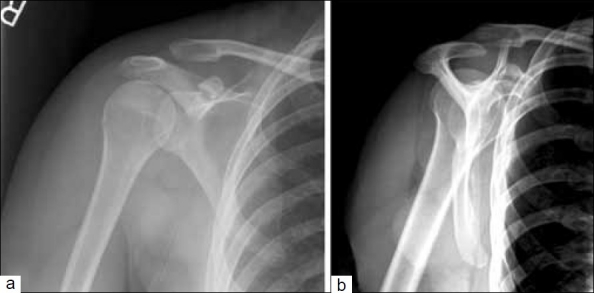
Anteroposterior radiograph (a) of the right shoulder demonstrates a coracoid fracture and type III acromioclavicular (AC) separation. It appears that the fracture is through the base of the coracoid process. The scapular lateral radiograph (b) redemonstrates the injury to the AC joint and visualization of the coracoid fracture

**Figure 2:**
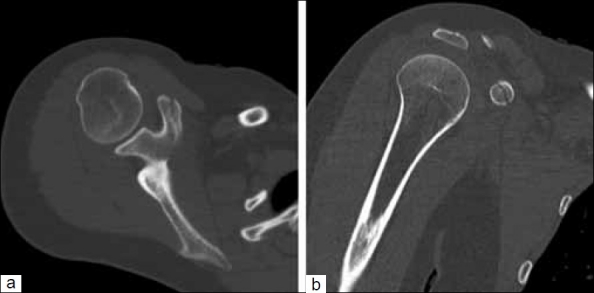
(a) Axial and (b) coronal computed tomography images demonstrate a sagittal plane fracture through the coracoid process

A 22-year-old right hand-dominant female collegiate athlete fell off a horse and landed directly on her right shoulder. She presented to the senior author’s clinic 1 week later, complaining of anterior shoulder pain. She denied any neurovascular complaints or prior history of injury to the shoulder. On an examination, she had ecchymosis and swelling around the AC joint, which was tender to palpation at the AC joint and coracoid process. Range of motion was limited due to pain. The neurovascular and cervical spine exams were normal. Radiographs demonstrated a Type III AC separation with a corresponding coracoid fracture. On x-ray, the fracture appeared to be located at the base of the coracoid [Figure 1]. However, the computed tomography (CT) scan showed that the fracture was not at the base but was in the sagittal plane through half of the coracoid [Figure 2 a and b].

**Figure 3:**
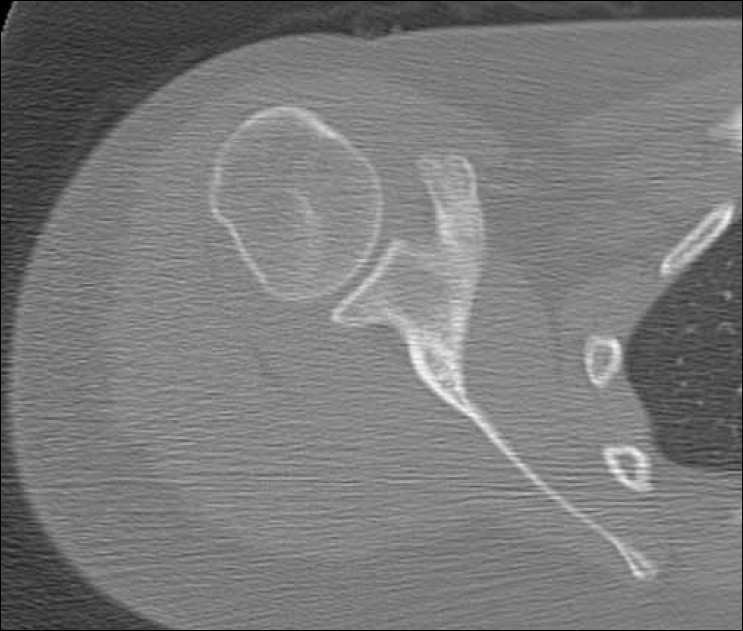
Axial computed tomography image demonstrates healing of the fracture at 3 months postinjury

The patient was placed in a sling and swathe, instructed to ice the shoulder and was given anti-inflammatory medication for pain. She was allowed to start gentle pendulum exercises 2 weeks post injury. After 6 weeks of immobilization, she was gradually progressed in her range of motion in all directions. A gentle rehabilitation program was started and once she was comfortable, a strengthening program progressed over the next 6 weeks. At 3 months, the patient was completely pain free and nontender to palpation. There was no AC instability on exam and she had full range of motion and strength. Repeat radiographs and CT scan demonstrated that the fracture was united [Figure 3 a and b]. The patient was released to return to full activities without restrictions.

## DISCUSSION

A fracture of the coracoid process associated with a complete acromioclavicular dislocation is a rare injury, with only 19 reported cases in adults in the English literature. This injury pattern was first described by Urist in 1941.[2] The mechanism of injury is the same for a Type III AC dislocation, except, instead of disrupting the coracoclavicular ligaments, a fracture occurs at the coracoid, which allows for vertical displacement of the clavicle.[1,3]

Diagnosing a coracoid fracture with a concomitant AC separation may be difficult.[4,5] The coracoid process initially projects upward and medially on the anterior aspect of the scapula and then it projects forward and laterally. Therefore, it appears shortened on a routine AP radiograph, making it difficult to identify a fracture. To accurately diagnose a coracoid fracture, special radiographs or a CT scan are necessary. Protass described a 30°–35° cephalad radiograph with the patient in a supine position.[6] CT can also better evaluate the coracoid process and provide the clinician with more detailed information regarding the location and extent of the coracoid fracture. In this case report, the radiographs appeared to show a transverse fracture through the base of the coracoid, but the CT scan confirmed that the fracture actually went horizontally through the coracoid. This information ultimately changed our treatment plan for the patient.

Selecting the appropriate treatment for this combined injury may be challenging as well. There are a number of different treatment options reported in the literature.[7] Of the 20 case series reported in adults in the English language, 12 were treated operatively[8–14] and eight were treated nonoperatively.[4,8,15–18] No clear guidelines for treatment exist currently.

Twelve of the 20 articles that reported a combined coracoid fracture and type III AC separation underwent operative fixation. The surgical procedures described are fixation of the AC joint, fixation of the coracoid and fixation of both the coracoid and the AC joint. Four cases were treated with open-reduction internal fixation of the AC joint alone. Three reports described AC joint fixation with K-wires and one with a Steinmann pin. Of the patients treated with K-wires, all reportedly had good results despite one patient with mild subluxation of the AC joint. The patient who underwent ORIF with a Steinmann pin did not have follow-up reported.

Four patients underwent fixation of the coracoid only. Zettas *et al*. reported fixing the coracoid process with a screw.[19] The patient had a good result, with the coracoid healing in 6 weeks. Ishizuka *et al*. described three patients who underwent a Dewar–Barrington procedure with good results.[13]

Three patients had fixation of both the AC joint and the coracoid process. One patient underwent screw fixation of the AC joint and the coracoid with a Dacron loop placed around the CC ligament. Two patients had K-wire fixation of the AC joint with screw fixation of the coracoid and reinforcement of the CC ligament with a synthetic loop. All three patients had good results with full range of motion, normal strength and no pain.

Of the surgically treated cases reported in the literature, only one study showed a poor result.[20] The patient experienced moderate pain and loss of function 1 year after surgery, which the authors attributed to poor postoperative rehabilitation. However, there were no details given about the patient or the type of surgical procedure the patient underwent. In addition, although these studies reported good results, the outcomes and complications of any type of pin fixation across the AC joint and in the shoulder have been well documented in the literature. Thus, it has yet to be determined the most appropriate way to address this constellation of injuries when surgery is indicated.[21,22]

Conversely, there are eight case reports of adults with a coracoid process fracture and an AC separation who were treated nonoperatively. Reasons for conservative management included multiple medical comorbidities, low physical demand and nondisplacement of the fracture. All reported satisfactory results.

The key to determining the appropriate treatment for these injuries is to recognize the fracture and determine its stability pattern.[14] Coracoid fractures most commonly occur at the base but have been described through the distal half of the coracoid and through the coracoid epiphysis.[5,23] Ogawa *et al*. classified coracoid fractures based on the anatomic relationship between the fracture site and the coracoclavicular ligament.[24] Type I fractures are behind the CC ligament, between the body of the scapula and the CC ligaments. These are typically associated with additional shoulder injuries such as AC joint dislocations. Type II fractures occur distal or in front of the CC ligaments – this is most commonly seen secondary to impaction by the humeral head or avulsion by the conjoined tendon. When a type I coracoid fracture is combined with an AC separation, there is a double disruption of the superior shoulder suspensory complex leading to a dissociation and destabilization of the scapuloclavicular connection.[25] Traditionally, surgical treatment has been recommended for type I injuries in order to avoid persistent instability and subsequent nonunion of the coracoid fracture. However, a type II fracture has stable CC ligament complex and therefore does not lead to a disruption of the SC connection. These injuries can be successfully treated without surgical intervention.

The case reported here is unusual because the patient is a female collegiate athlete with an unusual coracoid fracture pattern. This fracture pattern does not clearly fall into either a type I or type II injury according to the Ogawa classification. However, given the additional injury to the AC joint, it did present more like a potentially operative type I injury. Close CT evaluation did however show that instead of being fractured transversely through the base or the tip of the coracoid, the fracture occurred in the sagittal plane and did not extend into the glenoid fossa. This fracture pattern suggests that a portion of the CC ligaments were still attached to a part of the coracoid, maintaining a stable connection with the scapula like a type II fracture. Thus, this was viewed as a stable fracture pattern, nonoperative treatment was initiated and the patient went on to successful union without complications and no residual AC symptomatology within 3 months of the injury.

## CONCLUSIONS

A fracture of the coracoid process with a type III AC separation is a rare injury, with only 19 reported cases in adults. Both nonoperative and operative treatment of stable and unstable fracture patterns have been successful, making operative decision making difficult. Treatment of these injuries has traditionally been dependent on whether the injury pattern is stable, which is largely determined by the location of the coracoid fracture. This report adds another variant of this unique injury, treated successfully in a nonoperative fashion, to the literature and reemphasizes the importance of recognizing this unusual injury.

## References

[1] Mazzocca A, Arciero R, Bicos J (1997). Evaluation and treatment of acromioclavicular joint injuries. Am J Sports Med.

[2] Urist M (1963). Complete dislocation of the acromioclavicular joint. J Bone Joint Surg Am.

[3] Hak D, Johnson E (1993). Avulsion fracture of the coracoid associated with acromioclavicular dislocation. J Orthop Trauma.

[4] Carr A, Broughton N (1989). Acromioclavicular dislocation associated with fracture of the coracoid process. J Trauma.

[5] Combalia A, Arandes J, Alemany X, Ramon R (1995). Acromioclavicular dislocation with epiphyseal separation fo the coracoid process: Report of a case report and review of the literature. J Trauma.

[6] Protass J, Stampfli F, Osmer J (1975). Coracoid process fracture diagnosis in acromioclavicular separation. Radiology.

[7] Kim K, Rhee K, Shin H, Kim D, Shin H (2009). Displaced fracture of the coracoid process associated with acromioclavicular dislocation: A two-bird-one-stone solution. J Trauma.

[8] Montgomery S, Lloyd R (1977). Avulsion fracture of the coracoid epiphysis with acromioclavicular separation. J Bone Joint Surg.

[9] Smith D (1975). Coracoid fracture associated with acromoiclavicular dislocation. Clin Orthop Relat Res.

[10] Wang K, Hsu K, Shih C (1994). Coracoid process fracture combined with acromioclavicular dislocation and coracoclavicular ligament rupture. Clin Orthop Relat Res.

[11] Barentsz J, Driessen A (1989). Fracture of the coracoid process of the scapula with acromioclavicular separation. Case report and review of the literature. Acta Orthop Belg.

[12] Wilson K, Colwill J (1989). Combined acromioclavicular dislocation with coracoclavicular ligament disruption and coracoid process fracture. Am J Sports Med.

[13] Ishizuki M, Yamamaura I, Isobe Y, Furuya K, Tanabe K, Nagatsuka Y (1981). Avulsion fracture of the superior border of the scapula. J Bone Joint Surg.

[14] Eyres K, Brooks A, Stanley D (1995). Fractures of the coracoid process. J Bone Joint Surg Br.

[15] Bernard T, Brunet M (1983). Fractured coracoid process in acromioclavicular dislocations. Report of four cases and review of the literature. Clin Orthop Relat Res.

[16] Martin-Herrero T, Rodriguez-Merchan C, Munuera-Martinez L (1990). Fractures of the coracoid process: Presentation of seven cases and review of the literature. J Trauma.

[17] DiPaola M, Marchetto P (2009). Coracoid process fracture with acromioclavicular joint separation in an American football player: A case report and literature review. Am J Orthop.

[18] Lasda N, Murray D (1978). Fracture separation of the coracoid process associated with acromioclavicular dislocation. Clin Orthop Relat Res.

[19] Zettas J, Muchnic P (1976). Fractures of the coracoid process base in acute acromioclavicular separation. Orthop Rev.

[20] Wilber MC, Evans EB (1977). Fractures of the scapula. An analysis of forty cases and a review of the literature. J Bone Joint Surg Am.

[21] Motamedi M, Mortazavi S, Miresmaseeli S (2008). Migration of a broken Kirschner wire from an acromioclavicular joint into the neck: A case report. Eur J Orthop Surg Traumatol.

[22] Simovitch R, Sanders B, Ozbaydar M, Lavery K, Warner J (2009). Acromioclavicular joint injuries: Diagnosis and management. J Am Acad Orthop Surg.

[23] DeRosa G, Kettelkamp D (1977). Fracture of the coracoid process of the scapula. J Bone Joint Surg.

[24] Ogawa K, Yoshida A, Takahashi M, Ui M (1997). Fractures of the coracoid process. J Bone Joint Surg Br.

[25] Goss T (1993). Double disruption of the superior shoulder suspensory complex. J Orthop Trauma.

